# A brittle star is born: Ontogeny of luminous capabilities in *Amphiura filiformis*

**DOI:** 10.1371/journal.pone.0298185

**Published:** 2024-03-11

**Authors:** Constance Coubris, Laurent Duchatelet, Sam Dupont, Jérôme Mallefet

**Affiliations:** 1 Marine Biology Laboratory, Earth and Life Institute, Université catholique de Louvain, Louvain-La-Neuve, Belgium; 2 Department of Biological & Environmental Sciences, University of Gothenburg, Fiskebäckskil, Sweden; 3 IAEA Marine Environment Laboratories, Radioecology Laboratory, Monaco City, Monaco; Xinyang Normal University, CHINA

## Abstract

Bioluminescence is the production of visible light by living organisms thanks to a chemical reaction, implying the oxidation of a substrate called luciferin catalyzed by an enzyme, the luciferase. The luminous brittle star *Amphiura filiformis* depends on coelenterazine (*i*.*e*., the most widespread luciferin in marine ecosystems) and a luciferase homologous to the cnidarian *Renilla* luciferase to produce blue flashes in the arm’s spine. Only a few studies have focused on the ontogenic apparitions of bioluminescence in marine organisms. Like most ophiuroids, *A*. *filiformis* displays planktonic ophiopluteus larvae for which the ability to produce light was not investigated. This study aims to document the apparition of the luminous capabilities of this species during its ontogenic development, from the egg to settlement. Through biochemical assays, pharmacological stimulation, and *Renilla*-like luciferase immunohistological detection across different developing stages, we pointed out the emergence of the luminous capabilities after the ophiopluteus larval metamorphosis into a juvenile. In conclusion, we demonstrated that the larval pelagic stage of *A*. *filiformis* is not bioluminescent compared to juveniles and adults.

## Introduction

Bioluminescence is the production of visible light by living organisms thanks to a chemical reaction [1]. The ocean, the largest ecosystem on earth, shelters at least 80% of the known bioluminescent organisms [2, 3]. Recent phylogenetic works highlighted at least 104 apparitions of bioluminescence in the Tree of Life [4–6]. The function of bioluminescence in marine environments is often hypothesized but rarely experimentally demonstrated (*e*.*g*., *Vargula kuna*, *Harmothoe imbricata*, *Odontosyllis enopla*) [7–9]. Multiple ecological roles have been proposed, including attracting prey, escaping from a predator, or signaling with congeners [10]. Bioluminescence relies on specialized proteins named luciferases. Luciferases catalyze the oxidation of luminogenic substrates, commonly referred to as luciferins. This catalysis forms a transient intermediate, often a cyclic peroxide. This intermediate breaks down to produce oxyluciferin, emitting substantial energy as light [11, 12]. The luciferin and the luciferase can be grouped in a single unit called a photoprotein, where a co-factor might be needed to allow light emission [11, 13].

For decades, the bioluminescence phylogenic repartition was explained by the luciferase diversity, which was considered taxon-specific [10, 11, 14]. However, a recent study has identified 12 luciferase groups that, for some groups, co-evolved in non-related luminous organisms [15]. This phylogenomic analysis detected homologous luciferase sequences in distant phyla such as Cnidaria and Echinodermata [15, 16].

Among the luminous substrate, to date, four luciferins have been biochemically characterized in the marine environment: aldehydes in bacteria, tetrapyrroles in dinoflagellates, imidazolopyrazines known as the coelenterazine and the luciferin of copepod now called vargulin [11, 17]. The most widespread luciferin is the coelenterazine, an imidazopyrazinone compound (3,7-dihydroimidazopyrazin-3-one) found in at least nine phyla [14, 18, 19]. This large phylogenetic repartition is most likely due to a transfer of this luciferin through the food chain. Indeed, several studies have experimentally shown the coelenterazine trophic acquisition: one on a lophogastrid shrimp, *Gnathophausia inges* [20], a second on a jellyfish, *Aequorea victoria* [21], and more recently, on a brittle star, *Amphiura filiformis* [19]. The latter species is a burrowing filter-feeder echinoderm that emits blue light at 475 nm thanks to a coelenterazine-dependent luciferase homologous to the cnidarian *Renilla* luciferase [11, 16, 22]. As for some other luminous ophiuroids (*Amphipholis squamata*, *Amphiura arcystata*, *Ophiopsila californica*, *Ophionereis fasciata*, *Ophionereis schayeri*, *Ophiacantha aculeata*), *A*. *filiformis* flashes are elicited via a cholinergic control [22–29].

Most of the ophiuroids have a complex life history cycle alternating an adult benthic phase and a planktonic larval stage. *A*. *filiformis* is used as a model species around the world to study, among others, the extraordinary regenerative capabilities in echinoderms, for example, the recent release *A*. *filiformis* genome [30]. Moreover, this brittle star can be cultured in the laboratory throughout its life cycle [31, 32]. This aquaculture protocol allowed the study of the development of its nervous system and the evaluation of the impact of different stressors [33]. To date, the apparition of bioluminescence capabilities of *A*. *filiformis* remains unknown. Over the years, few studies have focused on the ontogenic apparitions of bioluminescence in marine organisms [34, 35]. Thomson et al., 1995, was the only study to measure the amount of coelenterazine at each developmental stage in the luminous shrimp *Systellaspis debilis*. This was monitored from the newly laid eggs to the juveniles, allowing the identification of a *de novo* synthesis of coelenterazine [35]. Similarly, through multigenerational maintenance of two ctenophore species, *Mnemiopsis leidyi* and *Bolinopsis infundibulum*, Bessho-Uehara et al. (2020) demonstrated the *de novo* synthesis of coelenterazine [36]. Other studies are more related to the ontogenic morphological development of light organs. Morphogenesis of luminous symbiotic organs was performed in organisms such as *Euprymna scolopes*, *Sagamichthys schnakenbecki*, and *Nuchequula nuchalis* [37–39], while few studies have been conducted on light organ development in intrinsic light emitters such as *Porichthys notatus*, *Etmopterus spinax*, and *Squaliolus aliae* [40–43]. Those few studies led to real comparison difficulties since they followed only proxies of luminous capabilities emergence during the ontogeny of organisms endowed with several luminous systems.

Knowing that *A*. *filiformis’* luminous abilities in adults depend on a continuous exogenous supply of coelenterazine [19]. As *A*. *filiformis* displays a two-stage life cycle, a larval pelagic stage and a benthic adult life form, larval coelenterazine acquisition could occur with the dietary shift between both. The parental transfer hypothesis was investigated to identify a potential coelenterazine and/or luciferase donation from the gonads to the progeny. Nevertheless, coelenterazine detection does not signify a species is luminous, as this molecule is found in non-luminous organisms [44]. Therefore, the luminous status of the larvae needs to be assessed. This study aims to determine from which ontogenic larval stage the brittle star can produce luminescence. Luminous capabilities of *A*. *filiformis* larval and juvenile stages were monitored through luminometric analyses. In parallel, the apparition of the luciferase expression was followed by immunodetection techniques. Results highlight that luminescence and luciferase expression occur after the larval metamorphosis and settlement.

## Material and methods

### Sampling and aquaculture protocol

*A*. *filiformis* individuals (Müller, 1776) were collected with an Eckman grab at a depth of 30–40 m in the Gullmarsfjord near the Kristineberg Marine Research Station (University of Gothenburg, Fiskebäckskil, Sweden) in August 2022 and 2023. The ophiuroids were carefully rinsed out of the mud, and intact specimens were placed in an aquarium with running seawater pumped directly from the adjacent fjord (12°C, 35 salinity). Based on Dupont et al., 2009, adult individuals with ripe gonads (white for testes and orange for ovaries) were isolated for spawning, fertilization, and larval cultures. Six males and females were directly microdissected under binoculars to extract the gonads. Gonads are transferred to Eppendorf tubes to be rinsed at least 4 times with filtered seawater (FSW) to remove body tissue. Gonad samples are either immediately used or frozen at -80°C. Juveniles were isolated in sediment cores collected by box coring at a depth of 40 m in September 2023. The 3 cm-topped oxygenated mud layer was carefully collected with a spoon and returned to the Kristineberg Marine Research Station. Mud was rinsed several times on a 62 μm sieve with deep sea water and examined under binoculars to collect juveniles among the meiofauna. Collected specimens were directly fixed in 4% paraformaldehyde (PFA) in phosphate-buffered saline (PBS: 123 mM NaCl, 2.6 mM KCl, 12.6 mM Na_2_HPO_4_, 1.7 mM KH_2_PO_4_, pH 7.4) for an hour, then rinsed in PBS before being stocked at 4°C until use for immunodetection protocol.

After overnight acclimation in the light, 8 males and 15 females were placed in FSW in the dark for 15 minutes until the release of sperm and eggs. About 1000 fertilized eggs at the two-cell stage were transferred to 25 one-liter aquaria at 14°C. The seawater was constantly aerated. After six days post-fertilization (dpf), larvae were fed daily with the red algae *Rhodomonas* sp. with a concentration in the culture of ~150 μg carbon per liter [32]. At various times (1, 5, 15, and 32 dpf), ophiopluteus larvae were transferred to Eppendorf tubes to be either immediately used, frozen at -80°C or fixed with 4% PFA-PBS to assess maximal light production (i.*e*., KCl depolarization experiments), the biochemical assays or the luciferase immunodetection protocol, respectively. Each sample was weighted before KCl depolarization experiments and luminometric assays. Due to the challenging process of obtaining enough larvae (*e*.*g*. larvae mortality, short reproduction season) for luminometric assays and optimizing the chance of getting metamorphosed and settled larvae (32 dpf), luminometric measurements at 25 and 28 dpf were not performed. A positive control was performed on the *A*. *filiformis* adult (S1 Table).

### Luminometric assay

Measurements of the light emission were carried out following Mallefet et al. (2020). An FB12 tube luminometer (Tirtertek-Berthold, Pforzheim, Germany) was kept in a dark room and calibrated using a standard 470 nm light source (Beta light, Saunders Technology, Hayes, UK). Light responses were recorded using FB12-Sirius PC Software (Tirtertek-Berthold). All data were standardized per unit of mass (g).

For KCl depolarization experiments, 500 μl of FSW containing gonads or 500 larvae (15 dpf) or 10 juveniles (32 dpf) was added to a tube. Light emission was triggered by injecting 500 μl of KCl solution (400 mM KCl, 52.3 mM MgCl_2_, 9.9 mM CaCl_2_, 27.7 mM Na_2_SO_4_, 20 mM Tris; pH 8.2). The total amount of light emitted (Ltot) was recorded and converted into quanta per gram of larval tissue (10^9^ q g^−1^). A control was performed on a non-luminous sympatric species, *A*. *chiajei*, using the same protocol [19].

At 32 dpf, 500 μl of FSW containing 10 juveniles were added to a tube. Light emission was triggered by injecting 500 μl of acetylcholine solution (2 mM ACh, 52.3 mM MgCl_2_, 9.9 mM CaCl_2_, 27.7 mM Na_2_SO_4_, 20 mM Tris; pH 8.2). Ltot was recorded and converted into quanta per gram of larval tissue (10^9^ q g^−1^).

For coelenterazine detection, 200 μl of cold methanol was added to an Eppendorf with gonads or at least 500 frozen larvae (1, 5, 15 dpf) or 10 juveniles (for 32 dpf) and crushed with micro-pestle. Then, 5 μl of the methanolic extract was placed into a tube filled with 195 μl of Tris buffer (20 mM Tris, 0.5 M NaCl; pH 7.4) and inserted in the luminometer. Afterward, 200 μl of *Renilla* luciferase solution with 4 μl of *Renilla* luciferase (Prolume Ltd., working dilution of 2 g l^–1^ in a Tris-HCl buffer 10 mM, NaCl 0.5 M, BSA 1%; pH 7.4) diluted in 196 μl of Tris buffer was injected into the luminometer tube. Ltot was recorded to calculate the coelenterazine content per gram of larval tissue (ng g^−1^), assuming that 1 ng of pure coelenterazine coupled with *Renilla* luciferase emits 2.52 × 10^11^ photons [11].

For the luciferase assay, 200 μl of Tris buffer was added to an Eppendorf with gonads or at least 500 frozen larvae (for 1, 5, 15 dpf) or 10 juveniles (for 32 dpf) and crushed with micro-pestle until a homogenized extract was obtained. Then, 20 and 40 μl of the extract were placed in two tubes with 180 and 160 μl Tris buffer, respectively. Each tube with the diluted luciferase solutions was put in the luminometer, and a solution with 5 μl of 1/200 stock of coelenterazine (1OD in cold methanol at 430 nm; Prolume Ltd, USA) diluted in 195 μl of Tris buffer was injected. Two measures of maximum light emission (Lmax) were recorded and averaged to calculate the maximal light decay rate corresponding to the luciferase activity, expressed in 10^9^ quanta g^−1^ s^−1^ [11]. For all measurements, at least 6 replicates were done.

### Luciferase immunolocalization

Fixed specimens, both larvae at 15, 25, 32 dpf, and meiofauna-collected juveniles, were rinsed three times in PBS (pH 7.7) and then blocked for 2 hours in PBS with 6% bovine serum albumin (BSA) and 2% Triton X100 at RT. *Renilla* luciferase antibody (GTX125851, Genetex, [16, 45]) was diluted 1:500 in PBS containing 6% BSA and 1% Triton X100. After overnight incubation (at room temperature), larvae were rinsed at least six times in PBS-1% Triton X100 and then incubated in a 1:500 dilution of Alexafluor 594 conjugated antirabbit (A11037, Thermofisher Invitrogen) in PBS containing 1% Triton X100 and 6% BSA. After incubation (2 hours, RT), larvae were rinsed six times with PBS-1% Triton X100. Specimens were mounted in Mowiol (Mowiol^®^ 4–88, Sigma) and examined using a Leica confocal microscope. For each stage, immunodetection experiments were performed on at least three specimens. A positive control experiment was performed using the same protocol on adult *A*. *filiformis* arms (S2 Fig). Besides, a control was performed by omitting the primary antibody to ensure the absence of unspecific binding of the secondary antibody.

### Statistical analysis

All the statistical analyses were performed with R Studio (version 2023.06.1, 2022, Posit Software, USA). Variance normality and equality were tested by the Shapiro–Wilk test and Levene’s test, respectively. When these parametric assumptions were met, an ANOVA coupled with Tukey’s test was used to test the effect of time post-fertilization on the tested parameters.

When log transformation does not allow reach normality and homoscedasticity, non-parametric Kruskal–Wallis ANOVA and Wilcoxon multiple comparisons test were used to assess the significant difference between different times post-fertilization. Each difference was considered to be significant at a minimum p-value <0.05. Values were graphically illustrated with mean and standard error of the mean (s.e.m). In addition, spearman correlations were performed between the luminometric measurements and developmental stages (S2 Table).

## Results

### *Amphiura filiformis* ontogenic development at 14°C

**Fig 1:**
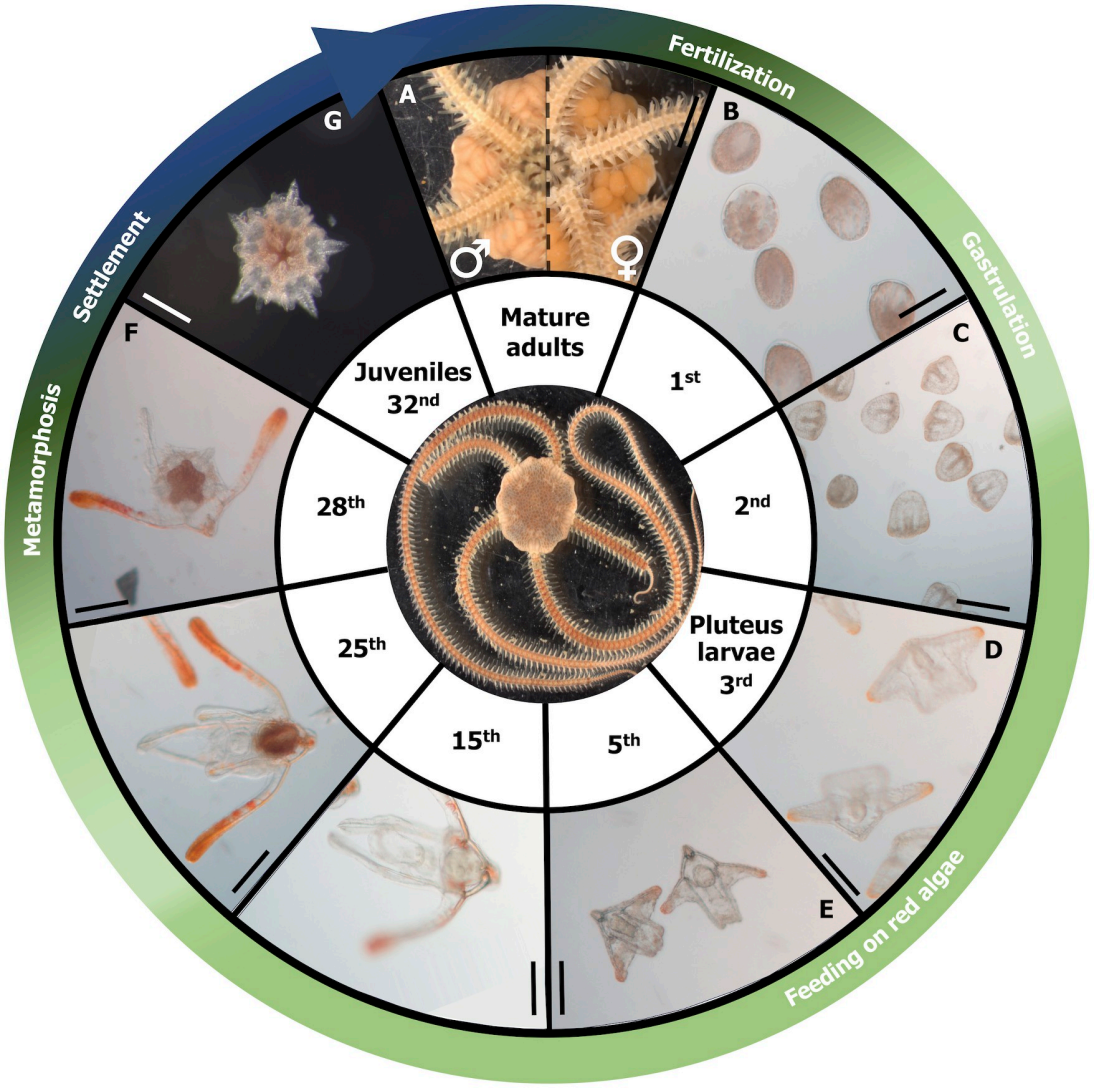
Reproductive cycle of *Amphiura filiformis* at 14°C. A: sexually mature adults (half-left male, half-right female), B: blastula, C: prism, D: early ophiopluteus, E: ophiopluteus, F: late ophiopluteus with larva rudiment, G: juvenile. The complete development of the brittle star *Amphiura filiformis* from the embryo to the juvenile takes around 30 days at 14°C. Scale bars represent (A), 500 μm, (B-C), 30 μm, (D), 40 μm, (E-F) 50 μm, (G), 80 μm. The color gradation from green (presence of coelenterazine) to blue (emergence of luminescence) on the arrow represents the evolution of luminous capabilities.

Mature individuals showed ripe gonads with distinguishable colors, white for males and orange for females (Fig 1A). On the first-dpf, *A*. *filiformis* embryos measured around 100 μm (Fig 1B). A prism shape corresponding to the late gastrula stage can be observed on the 2^nd^ dpf (Fig 1C). This stage is associated with the apparition of the digestive tract and the anus formation. On the 3^rd^ dpf, the ophiopluteus formation is characterized by a tripartite gut development and arms supported by calcareous spicules (Fig 1D and 1E). Along the ontogeny, the ophiopluteus gets flattened dorsoventrally. On the 28^th^ dpf, the pentameric development can be discerned with a remaining larva structure (Fig 1F). The metamorphosis and the settlement of the juvenile started at 32 dpf (Fig 1G). At this early settlement stage, the juveniles present a well-formed disc with five arms with one-to-two articles. *Rhodomonas* culture was tested to be coelenterazine-free (± 0.00002 ng g^-1^, n = 3) as a control.

### Luminous capabilities

**Fig 2:**
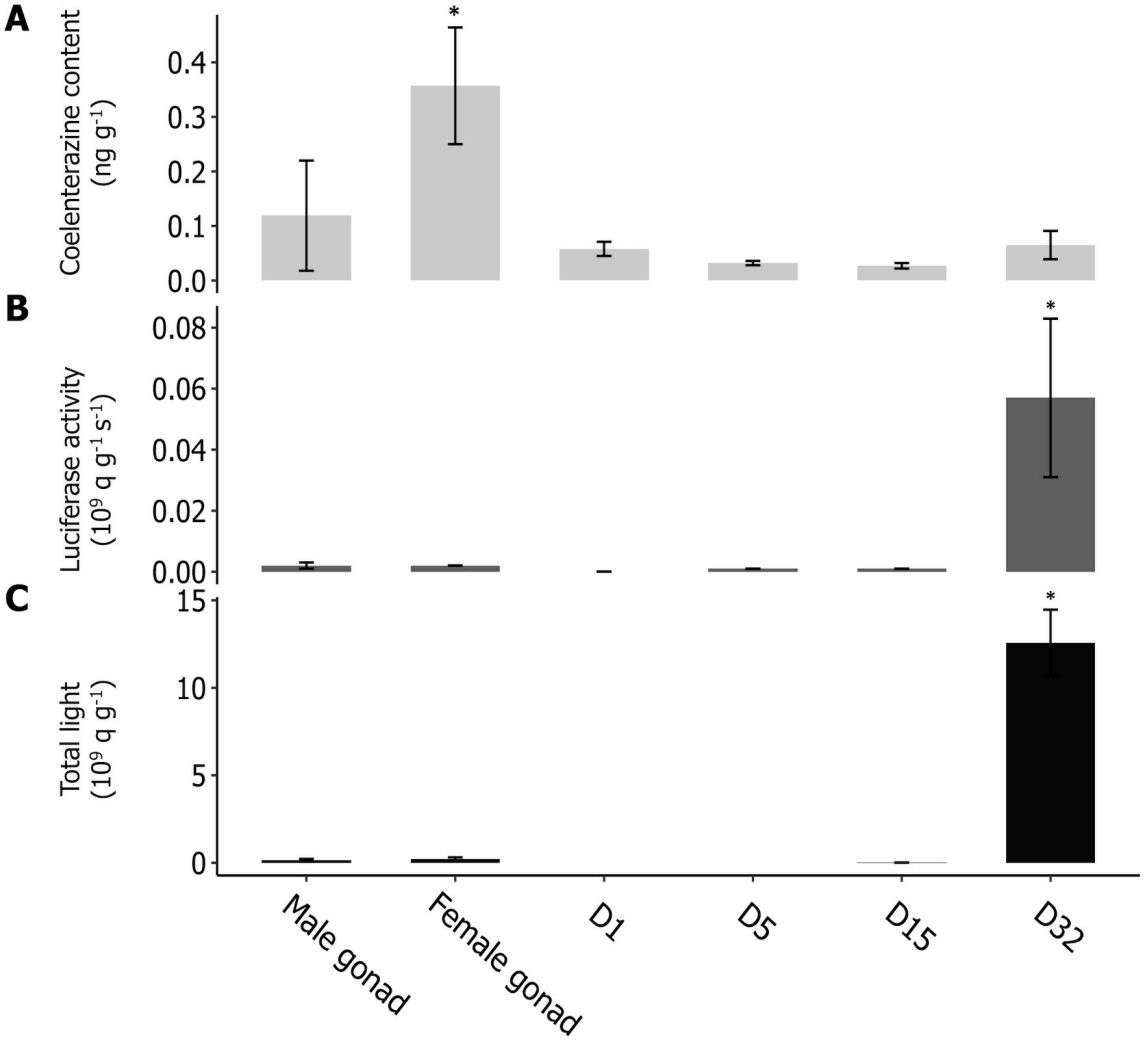
Luminescence capabilities of *Amphiura filiformis* larval stages from male and female gonads to 32 days post-fertilization (dpf). (A) Coelenterazine content (ng g^-1^) is significantly higher in the female gonads, (B) Luciferase activity (10^9^ q g^-1^ s^-1^) raised at 32 dpf, (C) Total light emission (10^9^ q g^-1^) with KCl stimulation (10^9^ q g^-1^) increased significantly 32 days after fertilization. Values are expressed as mean ± s.e.m. Asterisks indicate statistical differences between each larval stage with n≧6 (*i*) Kruskal–Wallis ANOVA and Wilcoxon multiple comparisons for coelenterazine content and total light emission, (*ii*) ANOVA and Tukey’s test for the luciferase activity; *P<0.05.

Two different patterns were observed for the different measured parameters. First, coelenterazine was detected at each ontogenic stage. It appeared significantly higher in the gonads, especially in ovaries (Kruskal–Wallis ANOVA and Wilcoxon, n≥6, Fig 2A), with a mean coelenterazine content of 0.36 ± 0.10 ng g^−1^. The mean coelenterazine amount measured in the juveniles was evaluated at 0.06 ± 0.02 ng. g^−1^. A typical response curve of coelenterazine assays is illustrated for 32 dpf juveniles in S1A Fig.

No correlation was observed between the coelenterazine content and the ontogenic stage (S2 Table).

Then, the luciferase activity and the total light increased significantly 32 days after fertilization (ANOVA and Tuckey, Kruskal–Wallis ANOVA and Wilcoxon, n≥6, Fig 2B and 2C). The luciferase activity and the total light emission increased respectively 120 and 50-fold at 32 dpf compared to levels observed in gonads. With a mean luciferase activity and total light emission after 32 days of 0.05 ± 0.02 10^9^ q g^−1^ s^−1^ and 12.57 ± 1.89 10^9^ q g^−1^, respectively. A positive correlation was detected during the ontogenic stages for the luciferase activity and the total light emission measurements (S2 Table). Moreover, the total light emission of juveniles at 32 dpf was statistically higher than the non-luminous control, *A*. *chiajei* (n = 6, P<0.05).

Acetylcholine injection at 32 dpf triggers light emission with an average light emission value (n = 6) of 0.12 ± 0.04 10^9^ q g^−1^. An example of the observed acetylcholine response curve for 32 dpf juveniles is shown in S1B Fig.

### Apparition of luciferase expression

**Fig 3:**
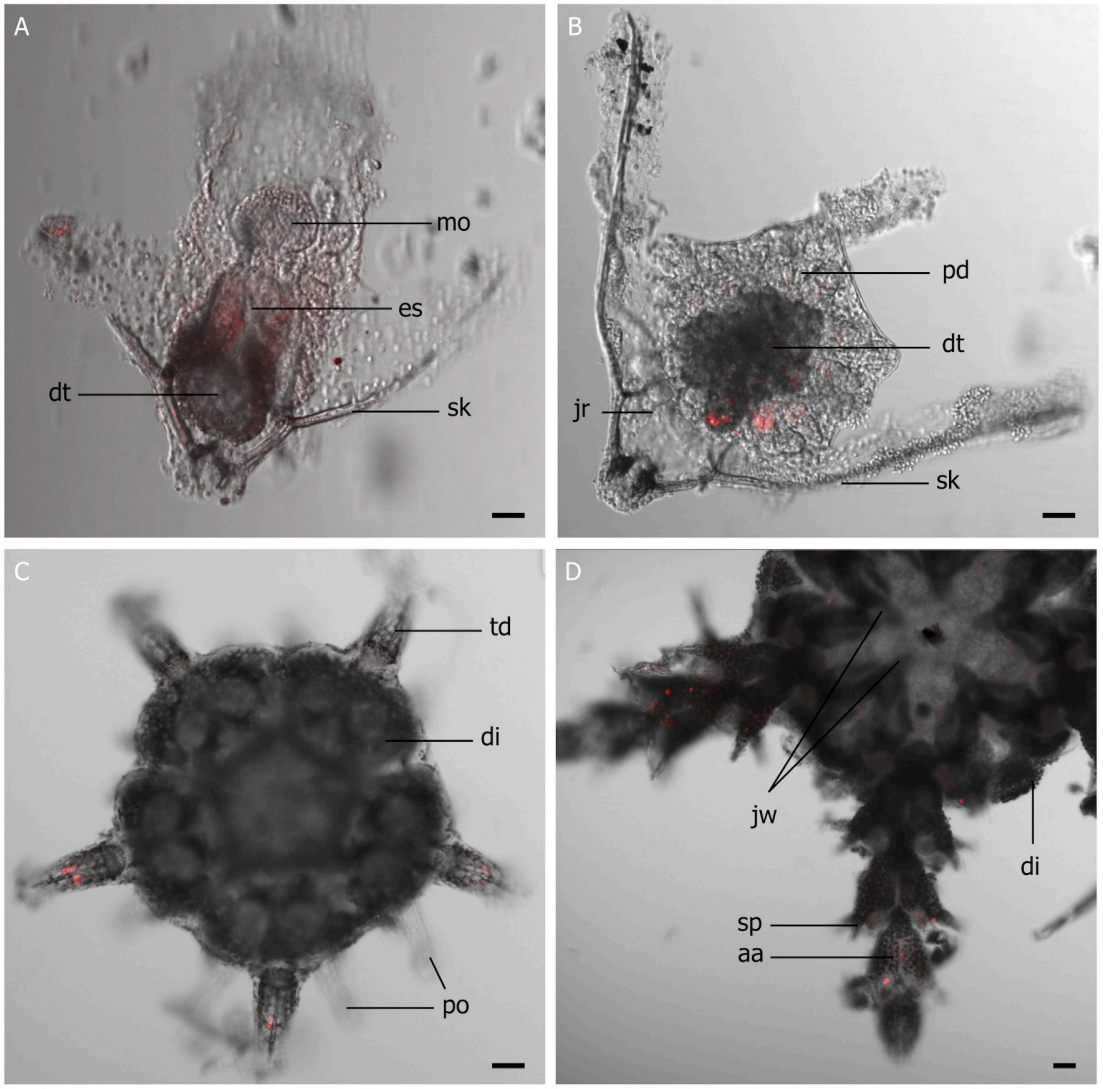
Immunolocalization of the *Renilla*-like luciferase (in red) in *Amphiura filiformis* larval stages; (A) 15 days, (B) 25 days, (C) 32 days post-fertilization, and (D) juveniles. aa, arm article; di, disk; dt, digestive tract; es, oesophagus; jr, juvenile rudiment; jw, jaws; mo, mouth opening; po, podia; pd, pentameric development; sk, skeleton rod; sp, spine; td, tip of the developing arm. Scale bar: 40 μm.

Knowing that *A*. *filiformis* uses a bioluminescence system based on a coelenterazine-dependent *Renilla*-like luciferase (up to 44% amino acid identity), a protocol with an antibody targeting the *Renilla reniformis* luciferase sequence was developed [16]. The whole mount *in toto* immunohistofluorescence revealed no labeling for the ophiopluteus larvae at 15 dpf (Fig 3A). Immunolabeling appeared faintly at 25 dpf during the metamorphosis from ophiopluteus larvae to juvenile, in the form of a pentaradiate marking, where the first articles of the future arms will be formed (Fig 3B). Finally, a strong immunodetection was observed in newly settled juveniles’ developing spine-bearing arms at 32 dpf (Fig 3C). Similar labeling was visible in older settled meiofaunal juveniles with staining on the spines carried on the arm segment (Fig 3D). Controls without the primary antibody did not show any staining. A positive control using adult arms showed labeling within the arm spines.

## Discussion

We cultured larvae of *A*. *filiformis* from fertilization to development at 14°C, following the protocol previously described [32]. A prism-shaped embryo was visible only 2 days after fertilization and was followed by the ophiopluteus stage on day 3 with the growth of the post-oral arm buds. The digestive tract was discernable from the 5^th^ dpf. At day 10, ophiopluteus have a tract of axon associated with the larval digestive tract [32]. On the 28^th^ dpf, the apparition of the juvenile pentameric rudiment with the first podia was visible on the surface of the stomach. In juveniles, the neuronal structure is much more similar to the ones observed in adults, with a single segmental unit and a nerve tract that extends to the tip of the developing arm [46, 47]. Dupont et al., 2009 suggested that additional segmental units were added when the arms grow in length. Our observation of a cholinergic luminescence triggering at 32 dpf is consistent with the apparition of the nervous system within the juvenile developing arms [32]. The luminous capabilities of *A*. *filiformis* also appeared after the metamorphosis and the settlement at 32 dpf (Fig 1).

Coelenterazine has been detected throughout the *A*. *filiformis* ontogeny from male and female gonads to the juvenile stage without any exogenous supply of coelenterazine since *Rhodomonas* culture is coelenterazine-free. Similarly, coelenterazine has been extracted from the arm tissues of the adult in natural conditions, but the relative amount is more than 100 times greater, with the mean value reaching 6 ng g^-1^ [19]. In 2020, Mallefet et al. demonstrated a significant decrease in coelenterazine content in the arm tissue of individuals maintained in captivity with a coelenterazine-free diet over a long-term period. These results have exhibited the inability of this species to *de novo* synthesize this luciferin. Detecting a small amount of coelenterazine recorded in the non-luminous larvae and juveniles of *A*. *filiformis* suggests a potential parental transfer of coelenterazine before its dietary acquisition. Several studies have measured coelenterazine content in the gonads and the eggs of multiple luminous species and hypothesized a parental transfer of the luminous substrate [21, 48–50]. The relative amount of coelenterazine in *A*. *filiformis* gonads is lower than the one measured in the gonads of myctophids species (20 ng g^−1^) or the *Argyropelecus hemigymnus* eggs (2 ng g^−1^) [48, 49].

The light emission capabilities are synchronized with the significant increase of the luciferase activity, which appears only after 32 dpf. Comparatively, adults’ total light emission and luciferase activity are 1000 times higher than juveniles’ [19]. Developing eggs luciferase activity in *A*. *filiformis* shows lower values than the one measure in adults (69 10^9^ q g^-1^ s^-1^) or other species, such as the eggs of the teleost species *Argyropelecus hemigymnus* (2 10^9^ q g^−1^s^−1^) [49]. The immunodetection confirms the luciferase presence in juveniles with specific expression of the *R-Luc* luciferase in the tip of the developing arm. Congruently, luciferase expression was detected throughout photocyte cells spread within the arm’s spines and tips [16].

While formers and the present study go forward on the bioluminescence biochemical processes involved in the brittle star [16, 19], some knowledge gaps remain concerning the ecological function and exact physiological control mechanisms under light emission. The precise action pathways of the involved cholinergic receptors remain unresolved [25]. Similarly, the findings of thirteen transcripts coding for opsins were found in *A*. *filiformis* adults with the presence and localization of one specific ciliary-based non-visual opsin demonstrated in the spines [45, 51–53]. The colocalized luciferase expression with the non-visual opsin in the arm’s spines led to the hypothesis this opsin plays a role in a photoemission-perception process regulating the amount of light produced in *A*. *filiformis* [53]. The hypothesis of coevolution of light emission and perception is strongly suggested in marine bioluminescent organisms [43, 54–57]. The inability to produce light for the early pluteus stage follows the transcriptomic data showing that the early pluteus (64 hours post-fertilization) lacks photoreceptive proteins [52].

Further studies are needed to understand the ontogenic implementation of the cholinergic receptors associated with nervous control and the ciliary-based opsin expression during luminescence ability apparition.

## Supporting information

S1 Fig Typical curve obtained for the 32 dpf juvenile of *Amphiura filiformis* in luminometric analyses. (A) Coenleterazine typical curve. (B) Acetylcholine (ACh 1mM) characteristic curve.
(DOCX)

S2 Fig Immunolocalization of the Renilla-like luciferase (in red) in arm tissue from an *Amphiura filiformis* adult *po*, *podia*, *sp*, *spine*. Scale bar: 40 μm.
(DOCX)

S1 Table Luminescence capabilities of *Amphiura filiformis* adults (n = 30). Measurements of the coelenterazine content (ng. g^-1^), the luciferase activity (10^9^ q. g^-1^. s^-1^), and the light emission after KCl depolarization and cholinergic stimulation (q. g^-1^) in the arm tissue.
(DOCX)

S2 Table Spearman correlation between developmental stage and luminometric measurement. (DOCX)
