# Supplementary material for: A brittle star is born: Ontogeny of luminous capabilities in Amphiura filiformis
Source: PLoS One. 2024 Mar 11;19(3):e0298185. doi: 10.1371/journal.pone.0298185 (PMC10927081; doi:10.1371/journal.pone.0298185)

# Supporting information

**S1 Fig.** **Typical curve obtained for the 32 dpf juvenile of *Amphiura filiformis* in luminometric analyses**. (A) Coenleterazine typical curve. (B) Acetylcholine (ACh 1mM) characteristic curve.

A


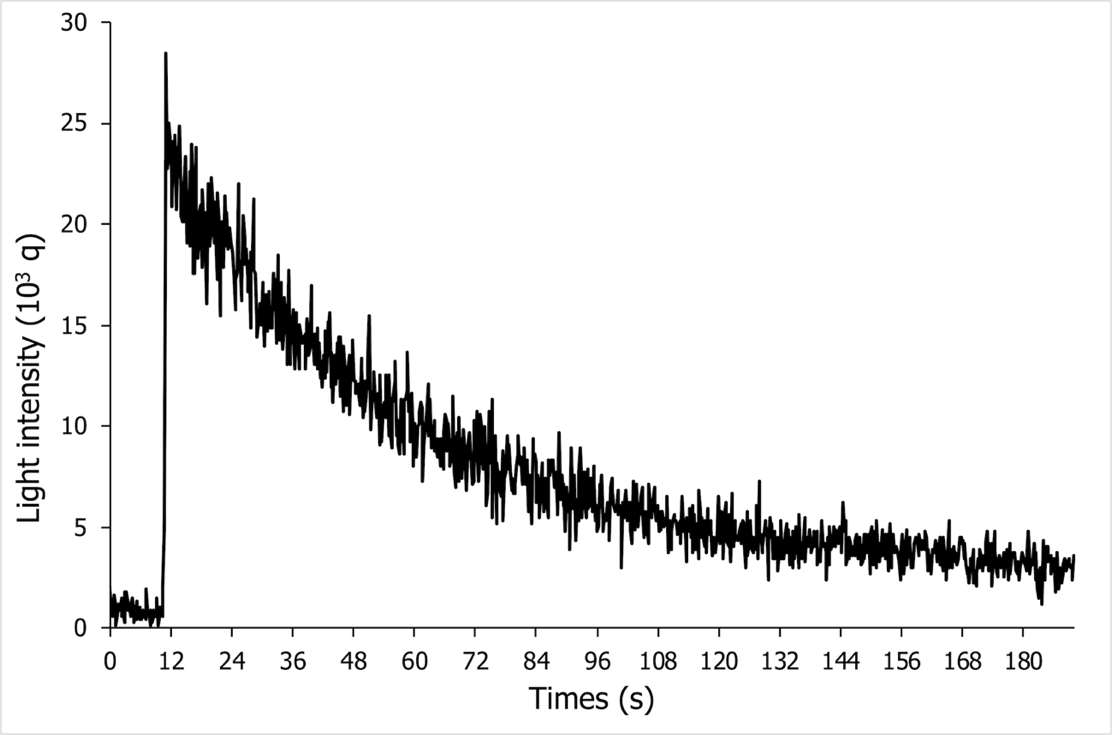


B


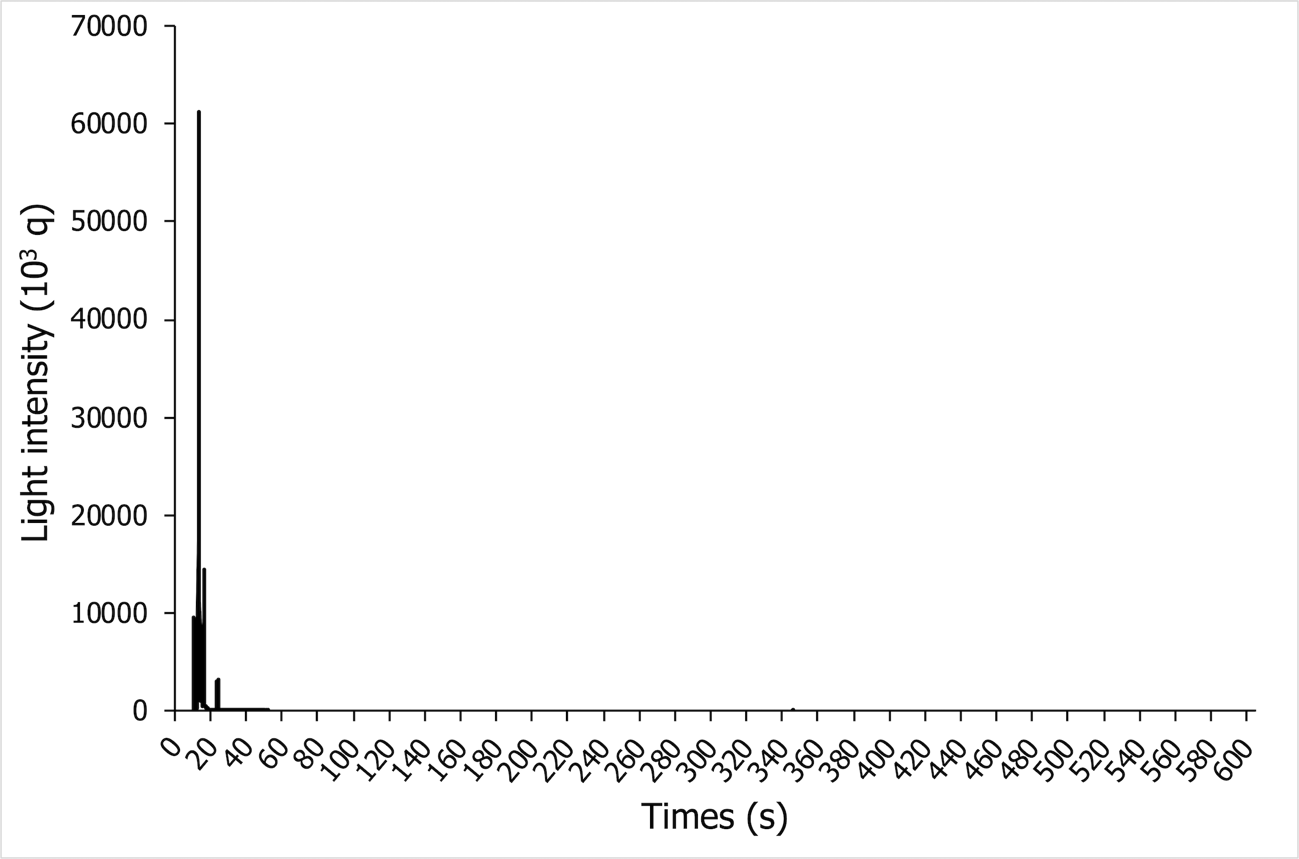

Supplement: S1 Fig — (A) Coenleterazine typical curve. (B) Acetylcholine (ACh 1mM) characteristic curve. (DOCX) [file pone.0298185.s001.docx]
