# Supplementary material for: A brittle star is born: Ontogeny of luminous capabilities in Amphiura filiformis
Source: PLoS One. 2024 Mar 11;19(3):e0298185. doi: 10.1371/journal.pone.0298185 (PMC10927081; doi:10.1371/journal.pone.0298185)
Supplement: S2 Fig — Scale bar: 40 μm. (DOCX) [file pone.0298185.s002.docx]

# Supporting information

*
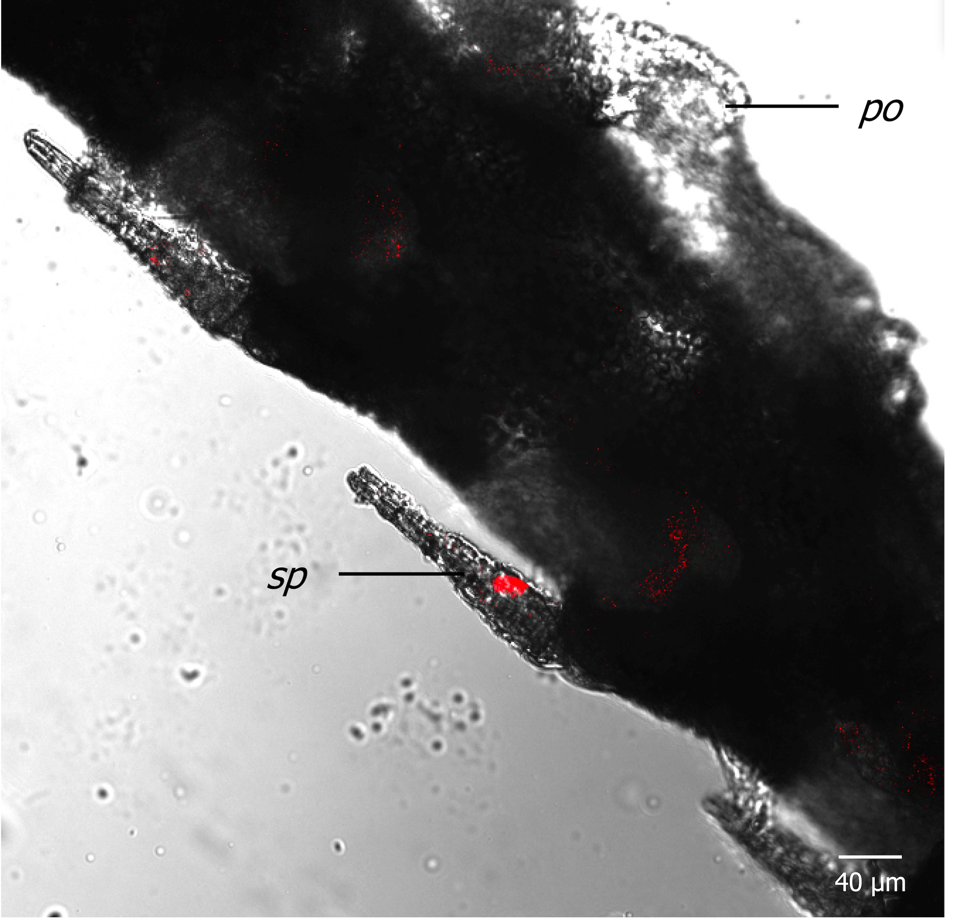
***S2 Fig. Immunolocalization of the Renilla-like luciferase (in red) in arm tissue from an *Amphiura filiformis* adult** *po, podia, sp, spine. Scale bar: 40 μm.*
