# Supplementary material for: A brittle star is born: Ontogeny of luminous capabilities in Amphiura filiformis
Source: PLoS One. 2024 Mar 11;19(3):e0298185. doi: 10.1371/journal.pone.0298185 (PMC10927081; doi:10.1371/journal.pone.0298185)
Supplement: S1 Table — Measurements of the coelenterazine content (ng. g-1), the luciferase activity (109 q. g-1. s-1), and the light emission after KCl depolarization and cholinergic stimulation (q. g-1) in the arm tissue. (DOCX) [file pone.0298185.s003.docx]

# Supporting information

**S1 Table Luminescence capabilities of *Amphiura filiformis* adults (n=30).** Measurements of the coelenterazine content (ng. g^-1^), the luciferase activity (10^9^ q. g^-1^. s^-1^), and the light emission after KCl depolarization and cholinergic stimulation (q. g^-1^) in the arm tissue.

|  | Mean value | s.e.m |
| --- | --- | --- |
| Coelenterazine content (ng. g^-1^) | 15.3 | 2.1 |
| Luciferase activity (10^9^ q. g^-1^. s^-1^) | 24 | 2 |
| Total light emission (q. g^-1^) | 8046 | 971 |
| Cholinergic stimulation (q. g^-1^) | 590 | 174 |
