# Supplementary material for: A brittle star is born: Ontogeny of luminous capabilities in Amphiura filiformis
Source: PLoS One. 2024 Mar 11;19(3):e0298185. doi: 10.1371/journal.pone.0298185 (PMC10927081; doi:10.1371/journal.pone.0298185)
Supplement: S2 Table — (DOCX) [file pone.0298185.s004.docx]

# Supporting information

**S2 Table Spearman correlation between developmental stage and luminometric measurement.**

|  | Rho |
| --- | --- |
| Coelenterazine content *VS* Developmental stages | -0.09 |
| Luciferase activity *VS* Developmental stages | 0.67 |
| Total light emission *VS* Developmental stages | 0.85 |
